# Glycoprotein 2 as a gut gate keeper for mucosal equilibrium between inflammation and immunity

**DOI:** 10.1007/s00281-023-00999-z

**Published:** 2024-01-03

**Authors:** Zhongwei Zhang, Izumi Tanaka, Rika Nakahashi-Ouchida, Peter B. Ernst, Hiroshi Kiyono, Yosuke Kurashima

**Affiliations:** 1 Department of Innovative Medicine, Graduate School of Medicine, Chiba University, 1-8-1 Inohana, Chuo-ku, Chiba, 260-8670 Japan; 2 Chiba University Futuristic Mucosal Vaccine Research and Development Synergy Institute (cSIMVa), Chiba, Japan; 3 Division of Mucosal Immunology, IMSUT Distinguished Professor Unit, The Institute of Medical Science, The University of Tokyo, Tokyo, Japan; 4 Division of Mucosal Vaccines, International Research and Development Center for Mucosal Vaccines, The Institute of Medical Science, The University of Tokyo, Tokyo, Japan; 5 Department of Human Mucosal Vaccinology, Chiba University Hospital, Chiba, Japan; 6 Department of Medicine, School of Medicine, Chiba University-University of California San Diego Center for Mucosal Immunology, Allergy and Vaccine (CU-UCSD cMAV), San Diego, CA USA; 7 Division of Comparative Pathology and Medicine, Department of Pathology, University of California, San Diego, CA USA; 8 Center for Veterinary Sciences and Comparative Medicine, University of California, San Diego, CA USA; 9 Future Medicine Education and Research Organization, Chiba University, Chiba, Japan; 10 HanaVax Inc., Tokyo, Japan; 11 Mucosal Immunology and Allergy Therapeutics, Institute for Global Prominent Research, Chiba University, Chiba, Japan; 12 Division of Clinical Vaccinology, International Research and Development Center for Mucosal Vaccines, The Institute of Medical Science, The University of Tokyo, Tokyo, Japan; 13 Institute for Advanced Academic Research, Chiba University, Chiba, Japan

**Keywords:** Mucosal immunology, Pancreas, Glycoprotein 2, Anti-GP2 autoantibodies, Inflammatory bowel disease, Pancreas-gut axis

## Abstract

Glycoprotein 2 (GP2) is a widely distributed protein in the digestive tract, contributing to mucosal barrier maintenance, immune homeostasis, and antigen-specific immune response, while also being linked to inflammatory bowel disease (IBD) pathogenesis. This review sheds light on the extensive distribution of GP2 within the gastrointestinal tract and its intricate interplay with the immune system. Furthermore, the significance of GP2 autoantibodies in diagnosing and categorizing IBD is underscored, alongside the promising therapeutic avenues for modulating GP2 to regulate immunity and maintain mucosal balance.

## Introduction

The gastrointestinal tract, the largest immune organ in the body, is crucial for the achievement and maintenance of immune homeostasis. The mucosal immune system (MIS) serves as a critical component in regulating both innate and adaptive immune responses, as it is constantly exposed to external dietary antigens and various beneficial and harmful microorganisms. Within this well-armed MIS, multiple anti-bacterial peptides are produced by enterocytes, goblet cells, Paneth cells, and plasma cells (e.g., Ly6/PLAUR domain containing 8 (Lypd8) [1], regenerating islet-derived 3-γ (Reg3γ) [2], mucin (3), α-defensins [3, 4], lysozyme C [5], phospholipases [6], C-type lectin [7], and immunoglobulin A (IgA) [8]).

In addition to the individual importance of the gut as a digestive and immunological organ, it is now being recognized as a pivotal link between various organs, the so-called organ axis, garnering increasing attention in medical research [9–11]. Numerous studies have also revealed a close relationship between the intestinal microbiota and digestive organ network of the intestine, liver, and pancreas [10, 12, 13]. For instance, gut microbiota (e.g., *Bacteroides*, *Clostridium*, *Escherichia*, *Egghertella*, *Fusobacterium*, *Lactobacillus*, *Peptococcus*, *Peptostreptococcus*, *Ruminococcus*) are indispensable components of bile acid metabolism [10]. When the homeostatic condition of intestinal microbes is disturbed, certain bile acid metabolites, such as deoxycholic acid (DCA), can accumulate, causing intestinal inflammation and even inducing gastrointestinal carcinogenesis [10, 14].

The pancreas is known for its essential role in the exocrine and endocrine functions of digestive enzymes. In addition to its fundamental role in digestion and maintaining certain hormone levels [15], the pancreas also plays a crucial role in forming and participating in the MIS-based intestinal barrier system [9]. The pancreas secretes a range of anti-microbial substances that are released into the gut lumen, including Reg2, Reg3β, Reg3γ, and secretory phospholipase A2 (sPLA2) [9, 12, 16, 17]. Furthermore, some enzymes (e.g., trypsin and lipase) derived from the pancreas indirectly protect the homeostasis of the gut microbiota [18, 19]. Pancreatic-derived trypsin plays a crucial role in activating other anti-bacterial proteins, such as Reg3α/γ and α-defensin, within the intestine [18, 20]. Pancreatic lipase indirectly inhibits potential intestinal inflammation and protects the intestinal barrier by increasing the abundance of probiotics, such as *Akkermansia muciniphila* and *Lactobacillus reuteri* [19]. Our recent research revealed the immunological importance of the pancreatic-gut axis for GP2-mediated intestinal balance between immunity and inflammation [12]. GP2 is abundantly secreted by the pancreas and plays a frontline protective role in preventing invasion of pathogenic bacteria into the host at the gut mucosal surface [12]. These studies further solidify the significant role of the pancreas as a frontline defender and a critical component of the MIS for maintaining healthy intestinal equalization by controlling immunity and inflammation.

GP2, initially discovered in the pancreas by MacDonald and Ronzio, is a member of the zona pellucida (ZP) domain protein family [21]. Its expression has been observed in certain multipotent pancreatic progenitor cells and pancreatic acinar cells [22, 23]. In acinar cells, GP2 is co-located on the glycolipid-enriched ectoleaflet (luminal surface) in apical secretory compartments, including secretory granules [24]. Within pancreatic acinar cells, GP2 is recognized as the predominant glycoprotein within zymogen granules (ZGs), comprising 25–40% of ZG membrane (ZGM)-associated glycoproteins [24, 25]. Over the years, extensive research has confirmed that GP2 is a ubiquitous glycoprotein in the digestive tract [12]. In addition to the pancreas, our previous studies have also demonstrated the expression of GP2 on professional antigen sampling cells known as microfold cells (M cells), which are located in the follicle-associated epithelium (FAE) of Peyer’s patches, a family member of MIS inductive tissues involved in the initiation of antigen-specific mucosal immunity [26, 27]. Notably, our study visually demonstrated the continuous presence of GP2 in the intestinal lumen (e.g., duodenum, jejunum, ileum, and colon) [12]. In the colon, we observed a pronounced co-localization of GP2 with commensal bacteria and the mucus layer. This layered distribution of GP2 adds an additional physicochemical barrier to the intestinal tract. However, in the small intestine (duodenum, jejunum, and ileum), since we did not stain for mucin, whether these GP2 proteins co-localize with the mucous layer as in the colon or are merely components of the intestinal contents or feces, requires further experiments to confirm.

## Molecular characteristics of GP2

According to the UniProt database (https://www.uniprot.org), GP2 is widely distributed among species, being found in mammals (e.g., humans, mice, rats, dogs, pigs, and bovines), poultry (e.g., ducks and geese), and even some fish (e.g., *Danio rerio*, *Salmo salar*, and *Carassius auratus*); this widespread distribution suggests the importance of conservation of GP2 across different animal species. In addition to this wide distribution, GP2 also has numerous isoforms [28, 29]. In *Homo sapiens* pancreas, four isoforms of GP2 have been identified and are believed to result from alternative splicing events [28, 29]. The four subtypes of GP2 exhibit pairwise similarities, with isoforms 1 and 2 sharing similarities, as do isoforms 3 and 4 [30, 31].

**Fig. 1:**
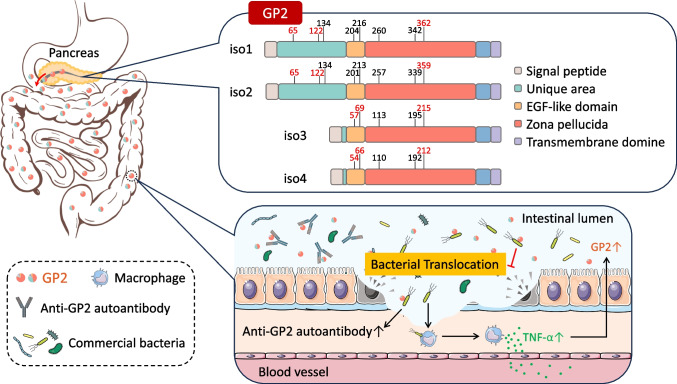
GP2 isoforms and their roles in IBD. In humans, GP2 is continuously produced by the pancreas and released into the intestine along with pancreatic juice. Human GP2 has four distinct isoforms, namely, the longer isoforms isoform 1 (537aa) and isoform 2 (534aa), as well as the shorter isoforms isoform 3 (390aa) and isoform 4 (387aa). The GP2 released into the intestine can bind to certain pathobionts, such as Escherichia coli , preventing their invasion of the mucosal tissue. In pathological conditions like IBD, the overgrowth of pathogenic bacteria can invade the mucosa, leading to inflammation, increased levels of TNF-α, and an upregulation of pancreatic GP2 production. However, simultaneously, translocated GP2-bound bacteria may act as immune enhancers, resulting in the loss of immune tolerance to GP2 in the body. This loss of tolerance leads to the production of autoantibodies against GP2, which neutralize the luminal GP2, thereby compromising GP2’s protective function

GP2 isoform 1 is the longest and most complete of all four isoforms that consists of 537 amino acids and several distinct domains. It begins with a signal peptide, followed by a unique long domain consisting of 197 amino acids, which contains three N-glycosylation sites, an epidermal growth factor (EGF)-like domain, and a zona pellucida (ZP) domain with three N-glycosylation sites (NCBI: NP_001007241.2, and predicted by SMART: http://smart.embl-heidelberg.de, and DTU Health Tech: https://services.healthtech.dtu.dk/) (Fig. 1). Finally, the protein has a C-terminal transmembrane domain that is removed in the Golgi apparatus upon attachment of the glycosylphosphatidylinositol inositol (GPI) anchor to the protein [30]. Among these domains, the EGF-like domain is recognized as a protein-protein binding domain [32]. The EGF-like domain is found in several ZP molecules, including ZP1, ZP2, ZP3, transforming growth factor receptor type 3 (TβRIII), and the urinary homologue of GP2, Tamm-Horsfall protein (THP) [32, 33]. The existence of an EGF-like domain may contribute to a protein’s functional role or aggregation, so this domain may be responsible for the propensity of GP2 to form tetrameric complexes.

Compared with isoform 1, isoform 2 lacks amino acids N179–181, resulting in a composition of 534 amino acids (NCBI: NP_001493.2). Isoform 3 is similar to isoform 4, both of which have relatively short isomers. Isoform 3 consists of 390 amino acids and lacks amino acids between N32 and 178 compared to isoform 1 (NCBI: NP_001007242.2), whereas the shorter isoform 4 lacks amino acids between N31 and 180 (NCBI: NP_001007243.2). The absence of these amino acids also leads to variations in the number of glycosylation sites among GP2 isoforms. The larger isoforms, 1 and 2, can be glycosylated at 8 sites, whereas the shorter isoforms (3 and 4) can only be glycosylated at 5 sites because of the absence of specific amino acids (predicted by DTU Health Tech: https://services.healthtech.dtu.dk/) (Fig. 1). The function of the GP2 isoforms might thus be influenced by alterations in their glycosylation sites due to the highly glycosylated nature of this glycoprotein [34].

## Biological journey of GP2 along the pancreas and intestine axis

As mentioned above, GP2 is a highly glycosylated protein comprising approximately 15% carbohydrates in dog [35] or rat [36]. This suggests that GP2 undergoes a series of significant and complex post-translational modifications (such as asparagine-linked glycosylation and intramolecular disulfide ligation [36, 37]) within the cell before entering the ZGs and being released outside the cell.

**Fig. 2:**
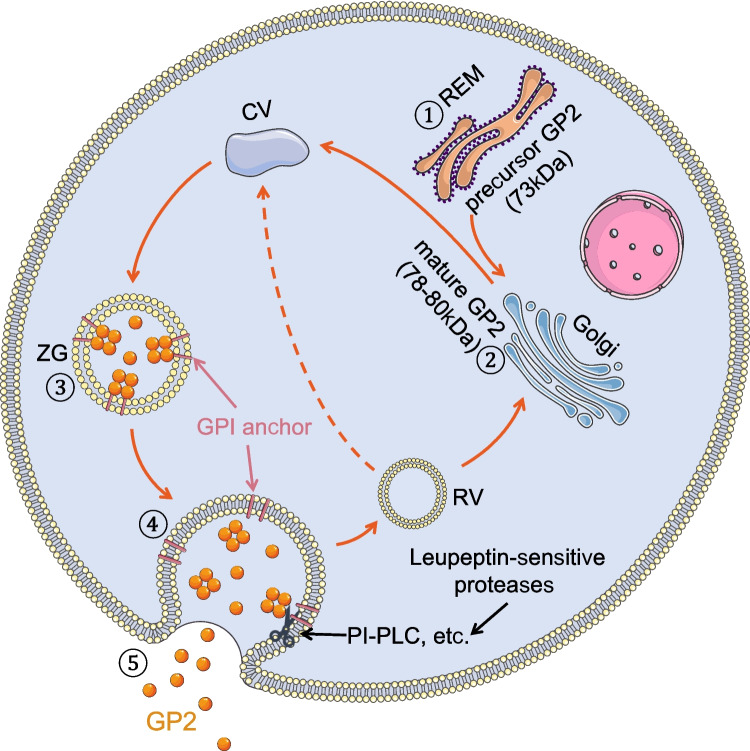
Biosynthesis and release of GP2 in pancreatic acinar cells. After the formation of the GP2 precursor glycoprotein, the high-mannose oligosaccharide chains are linked to the polypeptide backbone through the dolichyl phosphate pathway, which occurs within the rough endoplasmic reticulum. The precursor GP2 is then transported to the Golgi apparatus, where more complex trimming of the oligosaccharide chains and “complex sugar” glycosylation modifications occur. Subsequently, the protein becomes mature and is transported to the ZGs. Together with the ZGs, it reaches the apical plasma membrane, where it is selectively released from the membrane under the dual action of leupeptin-sensitive proteases and PI-PLC. REM: rough endoplasmic reticulum; CV: condensing vacuoles, ZG: zymogen (secretory) granules; RV: recycling vesicle; GPI: glycosylphosphatidylinositol; PI-PLC: phosphatidyl inositol-specific phospholipase C

Studies utilizing [^35^S] methionine pulse-chase labeling have provided valuable insights into the synthesis of GP2. Rat pancreatic acinar cells initially synthesize GP2 as an approximately 73-kDa precursor glycoprotein in the rough endoplasmic reticulum [36]. Five to six N-linked “high-mannose” oligosaccharide chains are linked to the polypeptide backbone through the dolichyl phosphate pathway. Given the use of different concentrations of tunicamycin (an N-glycosylation inhibitor) to treat isolated rat pancreatic acinar cells, the molecular weight range of the immunoprecipitated products was observed to be from 61 kDa (predicted as non-glycosylated GP2) to 73 kDa (high-mannose GP2) [36]. The precursor GP2 is then transferred to the Golgi apparatus (Fig. 2). Within the trans-Golgi network, which has a slightly acidic environment maintained by the H^+^ adenosine triphosphatase (ATPase), globular GP2 is released from the membrane and forms tetrameric complexes. These complexes are linked to sulfated proteoglycans, which have previously been recognized as components of the luminal surface of the ZGM [38, 39].

In the Golgi apparatus, N-glycosidically linked oligosaccharide chains undergo pruning, and “complex sugars” are added by Golgi glycosyltransferases. Consequently, a mature glycoprotein that weighs approximately 78–80 kDa is formed, and mature GP2 is then transferred to ZGs [36, 40–42] (Fig. 2).

Another study investigated GP2 synthesis in transfected canine kidney Madin-Darby Canine Kidney (MDCK) cells expressing both rat GP2 and human GP2, as well as in primary rat pancreatic cultures [43]. That study focused on the cleavage of amino acid ends during GP2 synthesis, as proteins can only be attached to glycosylphosphatidylinositol anchors after hydrolysis of these C-terminal signal peptides [44]. That group sequenced the amino acid terminus of human GP2 and identified a similar sequence at the end of GP2 in different human samples, Gly-Leu-Asp-Leu-Asp-Cys-Gly-Ala, indicating that the cleavage site of the GP2 protein occurs after Tyr38 (MERMVGSGLLWLALVSCILTQAS**A** [predicted signal peptide cleavage site] VQRGYGNPIEASS**Y** [potential cleavage site] GLDLDCGAPGTPE) during post-translational modification. Therefore, the cleavage site of the human GP2 signal peptide occurs after Ala24 [43]. The predicted sites aligned with the observed size changes in the molecular weight [43].

Similarly, the biosynthesis of rat GP2 involves complex processes [36]. The post-translational modification process of GP2 is completed within approximately 60 min [36]. Interestingly, GP2 undergoes further modification, leading to a smaller molecular form 2 to 4 h after synthesis [36]. This suggests that additional modification processes may occur after the initial post-translational modifications are completed.

GP2 exhibited a complex release process. Immunocytochemical observations have revealed that GP2 exists in two forms: a membrane-localized form (such as the endoplasmic reticulum, Golgi apparatus, ZGM, and plasma membrane) and a free protein form present within the interior of both ZGs and the acinar lumen [45, 46]. This dual localization indicates the journey of GP2 from its production and trafficking to its final release within the pancreatic acinar cells (Fig. 2).

The C-terminal GPI anchor of GP2 serves as the attachment point, anchoring it to the luminal leaflet of ZGM. Substantial evidence supports the notion that proteins possessing GPI structures have a greater propensity to localize to the apical plasma membrane in some polarized cells. This suggests that GP2 may travel along with the ZGs and localize at the glycolipid-enriched ectoleaflet within apical secretory compartments [41, 47, 48].

Studies using MDCK cells transfected with rat GP2 have shown that >95% of GP2 proteins are directed to the apical plasma [41, 47, 48]. Upon pancreatic secretion was stimulated such as by gastrointestinal hormones, ZGs move towards the cell membrane and undergo fusion, leading to GP2 release into the pancreatic duct (Fig. 2). This release is facilitated by the hydrolysis of the GPI anchor by endogenous phosphatidylinositol-specific phospholipase C (PI-PLC) [49–52]. However, it is important to note that the GP2-liberation since the plasma membrane is not solely dependent on phospholipase-mediated GPI-anchored cleavage. Instead, it is secondary to proteolytic hydrolysis; that is, the cleavage of GP2 from the membrane is primarily attributed to leupeptin-sensitive proteases rather than being a secondary consequence of phospholipase-mediated GPI-anchored cleavage [41]. This selective release mechanism has the significant advantage of allowing cells to selectively regulate the release of specific proteins, such as GP2, without releasing all GPI-anchored proteins simultaneously, considering the widespread existence of GPI-anchored proteins in vivo (Fig. 2). Furthermore, another study highlighted the fact that GP2, isolated from permeable ZGs, lacks the cross-reacting determinant (CRD) epitope [53]. It is widely recognized that the CRD epitope is an antigenic determinant of the inositol ring, which is exposed when GPI-anchored proteins undergo cleavage by PI-PLC [53]. Studies have also indicated that the release of GP2 is influenced by the pH conditions. GP2 cannot be secreted from the apical plasma membrane under acidic conditions [54, 55]. However, a fraction of GP2 within the ZGs may not be membrane-associated, with approximately 45% of GP2 being membrane-independent [52]. Upon cleavage and release, GP2 exhibits self-polymerizing properties, leading to the formation of macromolecules within the pancreas. These compounds are subsequently released along with digestive enzymes [51, 52].

The remarkably high concentration of GP2 in ZGs suggests its crucial role in ZGs’ function. Previous studies have also explored the significance of GP2 in ZG formation [42, 56, 57]. As discussed above, GP2 attaches to the ZGM through a GPI anchor (Fig. 2). Expanding on this understanding, Scheele et al. proposed that GP2 could form submembrane matrices with proteoglycans, potentially facilitating membrane sorting during granule assembly, ensuring stability during particle storage, and regulating zymogen transport from the apical plasma membrane following exocytosis [40].

Jacob et al. provided further support for this hypothesis by demonstrating the binding of amylase to GP2 in vitro under varying pH conditions, suggesting a potential role of GP2 in sorting aggregated secretory digestive enzymes into ZGs [58]. Colomer et al. also arrived at a similar conclusion by comparing the secretory form of GP2-GPI^-^ GP2 with pancreatic exocrine cell AR42J and pituitary endocrine cell AtT20 [42]. In addition, Scheele et al. posited that the liberation of the GP2/proteoglycan matrix from the apical plasma membrane might be a prerequisite for the internalization and subsequent reuse of ZGM [40]. Subsequent evidence supporting this hypothesis was presented by the same research group, which suggested that the alkaline environment within the acinar lumen modulates the release of the matrix from ZGs during exocytosis, leading to further endocytosis of the granules. Activation of PI-PLC is also involved in this process [54]. Several endocytosis-related proteins, such as pp60, p62yes, and caveolin, were also found to be abundant in GPI-anchored membranes and co-immunoprecipitated with GP2, suggesting that GP2 may regulate acinar cell endocytosis through a tyrosine kinase regulation pathway [59].

However, some other viewpoints suggest that GP2 may not be necessary for ZG formation [60]. Instances of ZG formation in the absence of GP2 have been previously reported. For example, the GP2 mRNA and protein expression do not occur until birth, but a significant number of ZGs are produced during embryonic development (15–21 days) in rats [61]. Partially differentiated pancreatic acinar cell carcinoma lines, specifically AR42J [61] and Reddy cells [62], were also found to be deficient in GP2. In addition, a study observed that ZGs in GP2 knockout mice displayed a normal morphology, number, and protein content, except for the absence of GP2, as confirmed by electron microscopy [60]. Therefore, further investigations are required to elucidate the underlying mechanisms [63].

Notably, our recent study provides compelling evidence of increased pancreatic GP2 expression in individuals with colitis [12]. In a mouse model of dextran sodium sulfate (DSS)-induced colitis, pancreatic GP2 levels were elevated. GP2 increase occurs without any apparent pancreatic pathological, histological (such as inflammatory cell infiltration), or physiological changes, such as altered pancreatic lipase expression [12]. Furthermore, elevated GP2 expression is specific to pancreatic cells, with a normal GP2 expression observed in follicle-associated epithelial cells of Peyer’s patches, which are also known to express GP2 [12].

To investigate the underlying mechanism behind the increased GP2 levels without obvious pathological changes in the pancreas, we investigated the synthesis, transport, and release processes of GP2. In particular, we focused on the examination of various guanosine triphosphate (GTPases) (e.g., Ras-Related Protein Rab3b, Rab6a, Rab8a, Rab27b, and Rap1a) and vesicular trafficking proteins (e.g., vesicle-associated membrane protein [Vamp] 2, Vamp3, Vamp8, syntaxin 3, and syntaxin 7) involved in the ZG transport system [12]. We also explored the expression of several enzymes that might cleave the GPI anchor, such as trypsin 5, phospholipases (Plcxd2, Plch1), and carboxypeptidases (Cpa1 and Cpa2). Data showed that the expression of the examined indicators did not change significantly. Nevertheless, based on our observations, we suggest that in cases of colitis, the increase in luminal GP2 may be attributed to de novo synthesis [12]. Further investigations revealed that the increased expression of GP2 in the pancreas was closely correlated with tumor necrosis factor-α (TNF-α), an inflammatory agent that is markedly elevated in cases of colitis [12]. Furthermore, our study successfully demonstrated that the released GP2 protein within the gut colocalizes with mucin, forming the primary line of defense against bacterial invasion [12].

Overall, the release of GP2 from ZGs involves complex mechanisms, including GPI anchor hydrolysis, proteolytic cleavage, disease, and pH sensitivity. These processes ensure the selective release of GP2.

## Multiple functions of GP2 in the digestive tract

The function of the GP2 protein within ZGs was extensively discussed in the previous section. However, considering the significant release of GP2 outside the cell after passing through the stages within ZGs, and the intricate nature of the human body, which typically avoids unnecessary processes, scientists have speculated that GP2 likely serves additional functions beyond ZGs. However, the precise role of GP2 in the pancreas and subsequent digestive tract has remained unclear [60].

Studies focused on the characterization of THP, a urinary counterpart of GP2, have provided valuable insights into the physiological functions of GP2 [53, 64]. THP is the predominant protein found in the urinary tract and originates from the renal tubular epithelial cells located in the ascending limb of Henle’s loop [53, 65]. In addition, THP has been found to regulate innate and adaptive immunity in the urinary tract [66] as well as enhanced the activity of monocyte proliferation and polymorphonuclear neutrophil phagocytosis [33, 67]. Studies have revealed that GP2 and THP share a common ancestral gene set, and divergence of genes between the urinary and digestive systems during development is the fundamental reason for their tissue specificity [68]. According to the GenBank database (https://www.ncbi.nlm.nih.gov/genbank/), the amino acid sequence of human GP2 exhibited 53% identity (percentage of positions in a pairwise alignment of 2 amino acid sequences with identical residues) and 85% similarity (percentage of positions in the alignment where the residues are either identical or have similar properties) with that of human THP [64].

Consequently, it appears that the functional characteristics of these two homologous proteins are partially preserved across both organ systems. Extensive research has been conducted on the anti-microbial function of THP in the urinary system [69, 70]. THP has been found to bind to type 1 fimbriae of *Escherichia coli* in the urinary tract, thereby preventing bacterial adhesion and invasion and subsequently reducing the likelihood of urinary tract infections [70–72].

Expression of GP2 on the apical surface of M cells located in the FAE of Peyer’s patches [26, 27], as well as its overexpression in biopsy samples taken from the colon of Crohn’s disease (CD) patients, has been extensively documented [12, 73–75]. In IBD pathophysiology, the intestinal microbiome plays a vital role in perpetuating inflammatory processes [76]. CD patients have been found to have a higher density of mucosal microbiota, particularly adherent bacteria interacting with Peyer’s patches, than healthy individuals [77, 78]. Building upon this observation, we made the assumption that GP2 might possess an immunobiological function that contributes to the creation of mucosal equilibrium between gut microbiota and the host, including prevention of adhesion and invasion of pathobionts. Our previous study showed that GP2 expressed on M cells was able to specifically bind to type 1 fimbrial adhesin (FimH) as the transcytotic receptor of bacteria [27]. FimH is a type 1 pili expressed in multiple pathogenic microorganisms, including *E. coli* and *Salmonella typhimurium* [79, 80]. Once M cells are anchored, GP2 attaches to the bacteria. Through trans-endocytosis, M cell-engulfed bacteria are captured by underlying dendritic cells, leading to the initiation of antigen-specific mucosal immunity [27, 73]. The absence of GP2 or FimH results in the reduction of antigen-specific serum immunoglobulin G (IgG) and gut IgA production by mucosal immunization targeting Peyer’s patches [27]. These findings suggest that GP2 is also involved in the induction and regulation of antigen-specific humoral immunity in both systemic and mucosal compartments especially expressed on Peyer’s patches. Furthermore, GP2 has been shown to enhance the *E. coli* phagocytic ability of monocytes [81].

As mentioned earlier, our recent study demonstrated that GP2, distributed extensively throughout the small and large intestines, is primarily derived from pancreatic acinar cells [12]. To further elucidate the immunobiological role of GP2 in vivo, pancreatic GP2-specific deficient mice were developed [12]. Following tamoxifen treatment, these mice exhibited a significant decrease in GP2 expression in the pancreas as well as in the luminal side of the digestive tract [12]. Notably, these GP2-deficient mice demonstrated heightened susceptibility to colon inflammation induced by DSS, resulting in more severe inflammatory responses than control mice [12]. We further investigated whether or not these pancreatic GP2 proteins could bind to FimH in a manner similar to anchored GP2 in M cells [12]. The pancreatic GP2 was capable of binding to FimH-positive *E. coli*. Furthermore, flow cytometry data demonstrated a notable increase in GP2-binding bacteria in inflamed GP2-deficient mice, which supports the crucial protective effect of GP2 [12] (Fig. 1).

Considering the interaction between GP2 and *E. coli*, we also explored whether GP2 might play a role in shaping the bacterial composition within the gut [12]. To investigate this notion further, we conducted 16S rRNA sequencing analysis to examine the composition of the intestinal commensal microbiota in both wild-type (WT) and GP2-deficient (GP2^−/−^) mice. The absence of GP2 did not appear to have a significant impact on the composition of the commensal microbiota at the phylum or genus levels. However, a closer examination revealed an increase in the populations of *Helicobacter* sp. and *Clostridium* sp., while the population of *Bacteroides acidifaciens* decreased in GP2^−/−^ mice. Nevertheless, the specific influence of these microbial changes on gut homeostasis and disease remains to be thoroughly investigated [82, 83]. Furthermore, we extended our investigation to young mice at the age of 2 weeks, and once again, we did not observe any substantial effects of GP2 deficiency on the composition of the gut microbiota [12].

In addition to assessing the impact of GP2 on the diversity of gut microbial species, we explored whether GP2 deficiency might lead to an increased bacterial load in the intestinal mucosa, which is particularly relevant since elevated mucosal bacterial numbers are often associated with colitis [84]. Using quantitative PCR, we measured the sizes of the total bacterial population (*Eubacteria*) and several specific bacterial populations, including *E. coli*, *Lactobacillus*, *Bacteroides*, *Helicobacter*, and *Proteus*, in the colons of mice [12]. The results indicated that in GP2-deficient mice, there was a notable increase in the population of mucosa *E. coli* compared to WT mice. This finding suggests that GP2 contributes to the regulation of the mucosal microbiota in the intestine, primarily through its influence on the population of *E. coli*. Consequently, GP2 deficiency may exacerbate the severity of colitis, considering the well-established association between increased *E. coli* levels and colonic inflammation [12]. Furthermore, our in vitro experiments indicated that binding to recombinant GP2 did not appear to affect bacterial growth.

To further understand GP2 protein binding to FimH, several studies have provided evidence that the glycosylation of GP2, particularly D-mannose [34, 85] and N-65 glycosylation [70], play critical roles. Furthermore, the binding capabilities of FimH and GP2 differ among variants/isoforms [34, 85]. GP2 isoforms 1 and 2 are considered to have a stronger affinity than short isoforms 3 and 4, possibly due to the presence of more glycosylation sites in these longer isoforms, providing additional asparagine residues and corresponding mannose residues, as discussed above [85]. However, according to the report from Derer et al. isoform 4, the dominant free-wandering GP2 isoform in the intestinal tract, binds mainly to the FimH of pathogens [29]. In addition, the different FimH variants and flow conditions (to simulate peristalsis in the natural gut) within the intestine have also been reported to influence the binding ability between GP2 and FimH [85]. Indeed, GP2 appears to have more than protective roles. In certain situations, there have been reports of GP2 binding to certain bacterial toxins, leading to its pathogenic effects. Studies have found that GP2 also exhibits binding affinity towards hemagglutinin A1 (HA) of botulinum neurotoxin, causing food-borne botulism [86]. HA-translocated GP2 disrupts the adherent junctions between M cells and enterocytes, impairing the epithelial barrier function [86].

Similar to the THP protein introduced above, GP2 is involved in more than just the gut-bacterial interaction and is thus capable of regulating the MIS. A previous study indicated that GP2 is recognized as the binding partner of scavenger receptors expressed on endothelial cell I (SREC-I), which are found on not only endothelial cells but also monocyte-derived dendritic cells (DCs) [87]. Given the widespread distribution of GP2 in the digestive tract, it is possible that DCs expressing SREC-I interact with GP2 or GP2 complexes, leading to internalization of GP2 and GP2 complexes by DCs. This suggests that GP2 may have a profound effect on the immune system, considering the pivotal role of DCs in both innate and adaptive immunity [87].

GP2 has also been associated with T cells. A previous study showed that GP2 expression in both peripheral blood mononuclear cells (PBMCs) and Caco-2 epithelial cells are regulated by activated human T cells and TNF-α inhibitors [81]. Intriguingly, treatment with recombinant GP2 showed significant effects on human intestinal epithelial, mucosal, and peripheral T cells, including reduced proliferation and apoptosis. GP2 also appears to regulate cytokine secretion, leading to decreased proinflammatory TNF-α and interleukin-17 (IL-17) levels and increased regulatory transforming growth factor β1 (TGF-β1) levels in PBMCs and freshly resected mucosal specimens from healthy volunteers or patients with non-inflammatory disorders [81]. In mucosal specimens, GP2 treatment resulted in decreased secretion of proinflammatory C-X-C motif chemokine ligand 8 (CXCL8) and increased secretion of regulatory TGF-β1 [81]. In addition, GP2-stimulate-intestinal epithelial cells demonstrated strong chemoattractant properties for T cells, which produced an effect similar to that of CXCL8 [81]. Based on these findings, Werner et al. proposed that GP2 might act as an anti-inflammatory and immunosuppressive agent in the intestinal mucosal system through its interaction with regulatory T cells [81].

## Anti-GP2 autoantibodies in IBD

The increased expression of GP2 in IBD has been extensively studied [12]. However, upregulation of GP2 also poses a risk of inducing autoantibodies [74]. Autoimmune responses were identified as essential triggers and perpetrators of IBD in patients with ulcerative colitis (UC) and CD, particularly in CD, as early as the 1950s and the 1970s, respectively [88]. Humoral autoimmune responses against pancreatic exocrine secretion have been observed in approximately 30% of patients with CD and 8% of patients with UC [89, 90]. It has also been noted that patients with pancreatitis are more susceptible to CD than the general population [91]. Pancreatic autoantibodies (PAB) exist in approximately 68% of CD patients with extraintestinal complications, such as idiopathic chronic pancreatitis [92–94]. Specific targets of PAB were identified in 2009, with GP2 being recognized as the primary autoantigen [73]. Furthermore, evidence indicates that, in addition to increased levels of pancreatic-synthesized GP2, both GP2 mRNA transcription and translation are elevated in the intestines of patients with CD [73, 74].

Two types of PAB were labeled based on the location of the indirect immunofluorescence signal [95]. The first type exhibits drop-like staining within the extracellular acinar lumen, whereas the second type shows speckled staining in the cytoplasm of acinar cells [95]. This distinction may arise from the two different states of GP2, which is released into the intestinal lumen alongside digestive enzymes and is also present in the ZGM of pancreatic acinar cells as a GPI-anchored membrane protein [52]. As mentioned above, the self-polymerization capability of GP2, resulting in the formation of high-molecular-weight structures [52], might generate new autoepitopes [96]. These factors contribute to the diversity and location specificity of PAB staining patterns.

The significance of anti-GP2 autoantibodies in the serological diagnosis of IBD has been thoroughly studied [74, 95, 97]. Studies have shown that patients with CD exhibit a higher prevalence of anti-GP2 autoantibodies than patients with UC. The incidence of anti-GP2 autoantibodies ranges from 21 to 45% in CD patients, 2 to 19% in UC patients, and 1 to 8% in healthy subjects [73, 97–99]. Studies have also shown that GP2 levels in the feces of CD patients are elevated compared with those in healthy individuals [12, 75]. Furthermore, patients with confirmed celiac disease also exhibit an autoimmune response to GP2 [94, 99]. Therefore, anti-GP2 autoantibodies can serve as a complementary diagnostic tool for stratifying patients. Importantly, when excluding patients with UC and celiac disease, the specificity of anti-GP2 autoantibodies for CD is approximately 98% compared to that for non-intestinal diseases [100].

The use of a novel enzyme-linked immunosorbent assay (ELISA) utilizing recombinant GP2 as a solid-phase antigen has revealed the existence of both IgG and IgA anti-GP2 autoantibodies [96, 98]. The levels of these two antibodies have been associated with more complex intestinal behaviors, such as ileal involvement, strictures, penetrating behavior, and surgery in patients with CD. Patients with structuring behavior and fibrostenotic complications exhibit elevated levels of anti-GP2 IgG, whereas individuals with penetrating diseases show reduced levels of anti-GP2 IgG [101–103]. Positive anti-GP2 autoantibodies have been linked to distinct patient characteristics, including an earlier onset, lower incidence of isolated colonic disease, and higher incidence of stenotic behavior anti-GP2 autoantibodies negative ones [104]. Furthermore, in patients with coexisting celiac disease, elevated levels of anti-GP2 IgA were observed, which correlated significantly with celiac disease-specific antibodies such as anti-tissue transglutaminase (anti-tTG) and anti-endomysial IgA antibodies [99]. In a study involving 174 individuals with celiac disease and 84 patients on a gluten-free diet (GFD), significantly increased levels of anti-GP2 IgA were found and showed a strong association with levels of endomysial antibodies (EMA) and anti-tTG IgA antibodies [105]. Moreover, this study notably revealed, for the first time, a significant correlation between the levels of anti-GP2 IgA and the degree of mucosal damage [105]. Interestingly, the loss of tolerance to GP2 appears to be transient and disease-related, as it disappears under GFD, a trend that has also been corroborated in another article [105, 106]. Notably, CD patients with pouchitis exhibit elevated levels of anti-GP2 IgA [107], and elevated levels of anti-GP2 IgA autoantibodies have also been detected in a minority of UC patients, particularly those with primary sclerosing cholangitis (PSC) as an extraintestinal manifestation [108, 109]. Further studies comparing anti-GP2 antibody titers in serum samples from independent cohorts of patients with PSC showed that anti-GP2 IgA autoantibodies were linked to a lower survival rate and an increased risk of developing cholangiocarcinoma, a type of bile duct cancer [110]. Another report indicated that the simultaneous detection of both anti-GP2 isoform 1 IgG and isoform 4 IgA was an effective diagnostic marker for PSC complicated with cirrhosis, exhibiting a sensitivity of 66.0% and specificity of 97.9% (Youden index: 0.64) [109].

The autoimmune response to GP2 is predominantly associated with the ileal site of disease, with notably lower levels of anti-GP2 autoantibodies detected in CD patients with colon involvement than in those with ileal lesions [98, 101, 111]. Even in UC cases, which generally have a reduced incidence of anti-GP2 autoantibodies, these antibodies can be detected in the serum and feces of patients who develop pouchitis after colectomy [107]. Furthermore, studies have shown a significant association between anti-GP2 autoantibodies and younger age (<16 years old) in CD patients [101]. In conclusion, a growing body of evidence strongly supports the association between the level of anti-GP2 autoantibodies and the specific clinical phenotypes of CD.

The presence of different isoforms of GP2 has raised the possibility that autoantibodies induced by specific isoforms have distinct implications in the diagnosis and pathogenesis of IBD. Research has shown that certain isoforms of GP2 are linked to the generation of autoantibodies and their effects on the pathophysiology of IBD. Overexpression of pancreatic-, intestinal M cell-, and L cell-derived GP2 isoform 4 (but not isoform 2), induced by TNF-α, in the gut of CD patients with ileocolic manifestations was shown to lead to the production of anti-GP2 autoantibodies [12, 29]. These autoantibodies increase the adherence and invasion of FimH-positive pathobionts within the intestine, thereby promoting the pathophysiology of IBD [29]. It was found that the detection of autoantibodies against the four isoforms of GP2 had diagnostic value in distinguishing between pediatric patients with UC and CD [31]. Pediatric patients with UC and CD with at least 1 positive antibody against the 4 isoforms of GP2 had a sensitivity of 54% and a specificity of 84%. Among CD patients, 42% had more than one type of anti-GP2 subtype of autoantibody. Anti-GP2 isoform 1 IgG and isoform 4 IgA are diagnostically valuable for the differential diagnosis of CD. The sensitivity of the anti-GP2 isoform 4 IgG to CD was 38%. However, anti-GP2 isoform 4 IgG serves as a relatively stable marker independent of disease activity over time only in 5% of UC (95% specificity) [31]. In addition, anti-GP2 isoforms 3 and 4 IgG showed high accuracy in differentiating between UC and CD [31].

Notably, research has demonstrated the stability of these anti-GP2 antibodies over an extended period. It was shown that only 5% CD patients changed the anti-GP2 isoform 4 IgA and IgG status, as well as other anti-pancreatic autoantibodies [112]. These findings emphasize the potential diagnostic and prognostic value of analyzing anti-GP2 autoantibodies, particularly in relation to specific isoforms, in differentiating between UC and CD and assessing disease activity and progression over time.

Furthermore, studies have implicated the microbial composition and the presence of specific bacteria in the disruption of immune tolerance towards GP2 and the subsequent development of GP2-intolerant autoimmunity. In general, the human body maintains immune tolerance to GP2, and the immune system is educated to suppress autoimmune responses against GP2. This tolerance is achieved, in part, through the regulation of GP2 as an autoimmune regulator-dependent gene in thymic epithelial cells, as reported by Rattay et al. [113]. This suggests that the loss of GP2-related tolerance may be related to the initiation and triggering of intestinal inflammatory responses. In our search, we also observed a significant increase in luminal anti-GP2 IgG autoantibody, consistent with human patients, in DSS-treated WT mice. Coupled with the observed increase in serum anti-*E. coli* antibodies in DSS-treated GP2^−/−^ mice, we determined that translocated GP2-binding bacteria may act as immune enhancers, promoting the development of an autoimmune response specifically targeting GP2 [12] (Fig. 1).

## The role of GP2 in other diseases and its potential as a therapeutic target

In addition to anti-GP2 autoantibodies in IBD, the role of GP2-related gene mutations in diseases has also been reported in recent years. Data analysis based on information from gnomAD (v3.1.2) (https://gnomad.broadinstitute.org/) has revealed a total of 94 synonymous mutations, 194 missense/in-frame indel mutations, and 35 predicted loss-of-function (pLOF) mutations have been found in the *GP2* gene. However, whether these mutations are pathogenic has rarely been studied. It was not until 2020 that a groundbreaking study based on a meta-analysis of three genome-wide association study (GWAS) datasets comprising 2039 pancreatic cancer patients and 32,592 controls from Japan confirmed the relatively pivotal role of *GP2* gene variants in pancreatic cancer [63]. This study analyzed a total of 7,914,378 SNPs across the whole genome and revealed three genome-wide significant loci associated with pancreatic cancer risk in this population: 13q12.2, 13q22.1, and the previously unreported 16p12.3 locus. Of particular note, the lead SNP at 16p12.3, namely, rs78193826 (a missense variant, C>T: p.V432M), exhibited a 46% increased risk of pancreatic cancer associated with each minor T allele. Furthermore, rs78193826 appears to be Asian-specific (especially East Asian), as the frequency of risk allele is around 8% in the East-Asian population and around 3% in South-Asian population but only 0.1% in populations of European ancestry [114]. Functional studies conducted in GP2-expressing pancreatic cancer cell lines demonstrated that rs78193826 may impact the activity of *KRAS*, a key driver of pancreatic cancer (with mutation frequencies >93%) [115]. These findings are particularly noteworthy as they unveil an Asian-specific *GP2* gene variant that was previously overlooked in Western populations. Additionally, considering that *GP2* variants are associated with a range of diseases and conditions beyond pancreatic cancer, including body mass index (BMI) (rs12597579) [116], type 1 [117] and 2 diabetes (rs117267808) [118], acute myeloid leukemia [119], and sleep quality [120], this study revealed the pleiotropic effects of GP2 variants, suggesting their potential roles in metabolic traits such as type 2 diabetes [63, 118]. In fact, in a comprehensive GWAS involving Japanese subjects, the top 3 16p12.3 locus SNPs, including rs78193826, showed significant associations with type 2 diabetes, glycated hemoglobin (HbA1c), and blood glucose levels [121]. Moreover, Mendelian randomization (MR) analysis hinted at intriguing associations between HbA1c levels and pancreatic cancer risk, suggesting the presence of shared genetic susceptibility [63].

GP2, as a multifunctional protein, is believed to have potential therapeutic applications beyond its role as a diagnostic tool. For instance, as previously mentioned, GP2 serves as a specific marker for pancreatic acinar cells [23, 122]. Building upon this foundation, Ameri and colleagues successfully isolated and differentiated functional β cells capable of responding to changes in glucose levels from GP2^+^ cells [123]. These newly generated β cells exhibited the ability to produce insulin in a glucose-dependent manner, similar to healthy β cells, holding tremendous potential for diabetes treatment [123].

Furthermore, GP2 has been shown to effectively prevent the infiltration of *E. coli* into the intestinal mucosa [12]. Even during colitis when pancreatic GP2 secretion increases, there are still more bacteria that remain unbound to GP2, which may contribute to the worsening of the inflammation [12]. Therefore, recombinant GP2 protein has emerged as a potential therapeutic approach for addressing dysbiosis during the onset of IBD. Building upon this, we inoculated GFP-labeled *E. coli* treated with recombinant GP2 protein into GP2^−/−^ mice [12]. The results demonstrated a significant reduction in the invasive capacity of the treated S17 in the mucosa, further validating the therapeutic potential of GP2.

Reducing the levels of anti-GP2 autoantibodies has also emerged as a potential therapeutic approach. Several studies have demonstrated the impact of microbes, particularly probiotics, on anti-GP2 autoantibody levels. It has been observed that untreated patients have higher levels of anti-GP2 autoantibodies than patients who have undergone treatment with probiotics, suggesting a potential role of microbial modulation in GP2-intolerant autoimmunity [107]. Further research is warranted to clarify the specific mechanisms underlying this process and explore potential therapeutic approaches targeting the modulation of the gut microbiome in the context of GP2-related autoimmune responses.

These exciting research findings provide a promising glimpse into the future of GP2’s therapeutic potential, successfully demonstrating the prospects of targeting GP2 in the treatment of autoimmune diseases and gastrointestinal disorders. However, some potential treatment modalities, such as recombinant GP2 protein formulations or interventions to inhibit autoantibody production, are still in the early stages of preclinical validation. Further research is needed to progress toward clinical validation, paving the way for more comprehensive studies and ultimately translating these innovative approaches into clinical practice.

## Conclusion

In conclusion, GP2 is a widely distributed protein in the digestive tract that plays multiple roles in the mucosal barrier system and has been implicated in IBD. Its involvement in IBD pathogenesis and potential as a serological diagnostic marker highlight its significance in this disease. However, as a highly glycosylated protein with multiple subtypes, the synthesis, post-translational modifications, membrane localization, and release of GP2 are highly complex, leading to discrepancies among researchers regarding its physiological functions. Therefore, further comprehensive research on GP2 is critically needed to fully understand its potential and pave the way for the development of innovative diagnostic and therapeutic approaches targeting GP2 in treating IBD.

## Data Availability

Data sharing not applicable to this article as no datasets were generated or analyzed during the current study.
