# Correction: Glycoprotein 2 as a gut gate keeper for mucosal equilibrium between inflammation and immunity

**DOI:** 10.1007/s00281-024-01002-z

**Published:** 2024-04-22

**Authors:** Zhongwei Zhang, Izumi Tanaka, Rika Nakahashi-Ouchida, Peter B. Ernst, Hiroshi Kiyono, Yosuke Kurashima

**Affiliations:** 1 Department of Innovative Medicine, Graduate School of Medicine, Chiba University, 1-8-1 Inohana, Chuo-ku, Chiba, 260-8670 Japan; 2 Chiba University Futuristic Mucosal Vaccine Research and Development Synergy Institute (cSIMVa), Chiba, Japan; 3 Division of Mucosal Immunology, IMSUT Distinguished Professor Unit, The Institute of Medical Science, The University of Tokyo, Tokyo, Japan; 4 Division of Mucosal Vaccines, International Research and Development Center for Mucosal Vaccines, The Institute of Medical Science, The University of Tokyo, Tokyo, Japan; 5 Department of Human Mucosal Vaccinology, Chiba University Hospital, Chiba, Japan; 6 Department of Medicine, School of Medicine, Chiba University-University of California San Diego Center for Mucosal Immunology, Allergy and Vaccine (CU-UCSD cMAV), San Diego, CA USA; 7 Division of Comparative Pathology and Medicine, Department of Pathology, University of California, San Diego, CA USA; 8 Center for Veterinary Sciences and Comparative Medicine, University of California, San Diego, CA USA; 9 Future Medicine Education and Research Organization, Chiba University, Chiba, Japan; 10 HanaVax Inc., Tokyo, Japan; 11 Mucosal Immunology and Allergy Therapeutics, Institute for Global Prominent Research, Chiba University, Chiba, Japan; 12 Division of Clinical Vaccinology, International Research and Development Center for Mucosal Vaccines, The Institute of Medical Science, The University of Tokyo, Tokyo, Japan; 13 Institute for Advanced Academic Research, Chiba University, Chiba, Japan

**Correction: Seminars in Immunopathology**

10.1007/s00281-023-00999-z

In this article the statement in the Funding information section was incorrect.

The incorrect statement is as follows:

**Funding** This work was supported by Japan Society for the Promotion of Science (JSPS) Grant-in-Aid for Scientific Research S (18H05280 to H. K., 23H02699 to Y. K.); JSPS Fund for the Promotion of Joint International Research (18KK0432 to Y. K.); Japan Agency for Medical Research and Development (AMED) (JP223fa627003, N.-O., Y. K., and H. K.); AMED Project Focused on Developing Key Technology for Discovering and Manufacturing Drugs for Next-Generation Treatment and Diagnosis (NeDDTrim) (JP21ae0121040 to H. K.), PRIME (20gm6010012h0004/20gm6210024h0001); Future Medicine Fund of Chiba University (to Y. K.); the Chiba University-UC San Diego Center for Mucosal Immunology, Allergy, and Vaccines (to Y. K., P. E., and H. K.); the Yamada Science Foundation (to Y. K.); and a 3M donation (to H. K.), JST SPRING, Grant Number JPMJSP2109 (to Z. Z.), and the Chiba University-UC San Diego Center for Mucosal Immunology, Allergy, and Vaccines (cMAV).

In the corrected statement the following was added:

and Japan Initiative for World-leading Vaccine Research and Development Centers (JP223fa627003 to H. K. and Y. K.); cSIMVA Vaccine Challenge Grants (to Z. Z.);

Futhermore, the below information was removed:

, and the Chiba University-UC San Diego Center for Mucosal Immunology, Allergy, and Vaccines (cMAV)

Additionally, there has been one minor addition.

The complete correct funding statement is as follows. **Bold is used to highlight new additions**.

**Funding** This work was supported by Japan Society for the Promotion of Science (JSPS) Grant-in-Aid for Scientific Research S (18H05280 to H. K., 23H02699 to Y. K.); JSPS Fund for the Promotion of Joint International Research (18KK0432 to Y. K.); Japan Agency for Medical Research and Development (AMED) (JP223fa627003, N.-O., Y. K., and H. K.); AMED Project Focused on Developing Key Technology for Discovering and Manufacturing Drugs for Next-Generation Treatment and Diagnosis (NeDDTrim) (JP21ae0121040 to H. K.), PRIME (20gm6010012h0004/20gm6210024h0001); **and Japan Initiative for World-leading Vaccine Research and Development Centers (JP223fa627003 to H. K. and Y. K.); cSIMVA Vaccine Challenge Grants (to Z. Z.);** Future Medicine Fund of Chiba University (to Y. K.); the Chiba University-UC San Diego Center for Mucosal Immunology, Allergy, and Vaccines **(cMAV)** (to Y. K., P. E., and H. K.); the Yamada Science Foundation (to Y. K.); and a 3M donation (to H. K.), JST SPRING, Grant Number JPMJSP2109 (to Z. Z.).

The original article has been corrected.

